# The Delivery Challenge in Neurodegenerative Disorders: The Nanoparticles Role in Alzheimer’s Disease Therapeutics and Diagnostics

**DOI:** 10.3390/pharmaceutics10040190

**Published:** 2018-10-17

**Authors:** Cristina de la Torre, Valentín Ceña

**Affiliations:** 1 Unidad Asociada Neurodeath, Universidad de Castilla-La Mancha, Almansa, 14, 02006 Albacete, Spain; kristina8cstm@gmail.com; 2 CIBERNED, Instituto de Salud Carlos III, 28031 Madrid, Spain

**Keywords:** Alzheimer’s disease, blood-brain barrier, nanoparticles, animal models

## Abstract

Alzheimer’s disease (AD) is one of the main causes of disability and dependency among elderly people. AD is a neurodegenerative disorder characterized by a progressive and irreversible cognitive impairment, whose etiology is unclear because of the complex molecular mechanisms involved in its pathophysiology. A global view of the AD pathophysiology is described in order to understand the need for an effective treatment and why nanoparticles (NPs) could be an important weapon against neurodegenerative diseases by solving the general problem of poor delivery into the central nervous system (CNS) for many drugs. Drug delivery into the CNS is one of the most challenging objectives in pharmaceutical design, due to the limited access to the CNS imposed by the blood-brain barrier (BBB). The purpose of this review is to present a comprehensive overview of the use of NPs as delivery systems for therapeutic and diagnostic purposes in models of AD.

## 1. Introduction

Dementia is one of the main causes of disability and dependency among people aged 65 and older all over the world [1]. It is characterized by a decrease in cognitive function, memory, thinking ability, reasoning, and learning caused by an abnormal aging of the central nervous system (CNS). This syndrome produces a great physical, psychological, sociological, and economic impact on the population, limiting the functional independence of the patient, which affects both the own patient and his/her social milieu. Dementia can be caused by different neurological diseases. However, Alzheimer’s disease (AD) causes between 60% and 70% of all cases of dementia, which converts it in a public health priority due to its prevalence and its impact on society [1].

AD is a neurodegenerative disorder characterized by a progressive and irreversible neuronal damage that was described for the first time by Alois in 1906 [2]. Since then, several studies have been conducted to identify the cause of AD and to describe precisely its pathophysiology to find an effective treatment to cure the disease. However, so far it has not been possible to decipher the precise process by which neurodegeneration occurs and therefore, only symptomatic treatments are available. Those treatments are based on: (i) the inhibition of the acetylcholinesterase enzyme (donepezil, galantamine, rivastigmine, tacrine), to restore the cholinergic function and (ii) antagonism of *N*-methyl-d-aspartate receptors (NMDAr), to reduce the excitotoxicity caused by an excess of glutamate (memantine) [3]. These treatments promote a relative improvement of cognitive ability and slow down the neuronal damage, but all of them are insufficient to halt the advance of AD, which is the reason why new effective treatments, capable of coping with this disease, are required.

## 2. Main Mechanisms Involved in AD Pathophysiology

**Figure 1:**
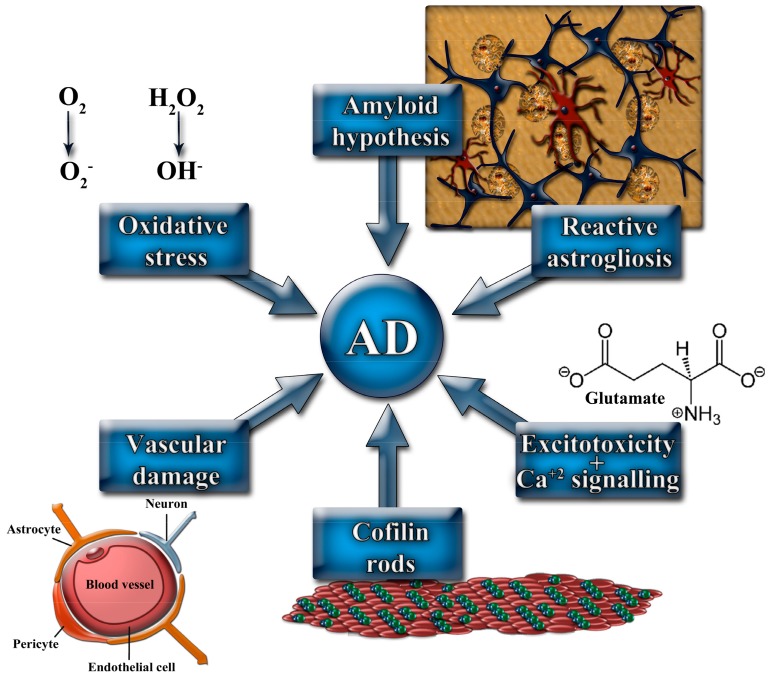
Main mechanisms involved in the pathophysiology of Alzheimer’s disease (AD).

The complexity of AD pathophysiology has led the scientific community to propose several mechanisms that might contribute to the genesis of this disease (Figure 1).

### 2.1. The Amyloid Cascade Hypothesis

**Figure 2:**
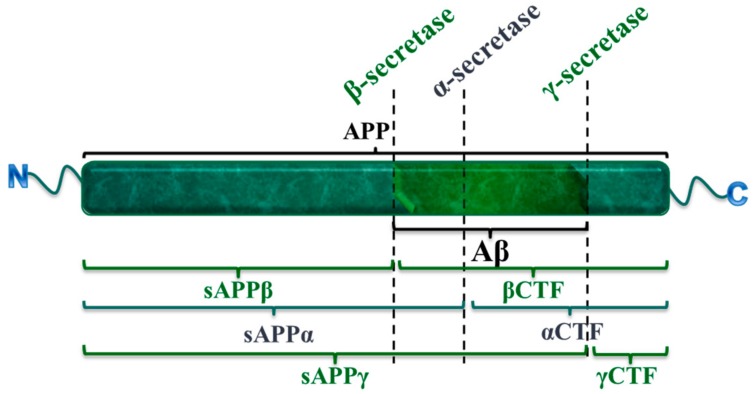
The different cleavage sites of the secretase complex and the APP-fragments (β-amyloid protein precursor fragments) that each secretase yields: The amyloidogenic processing (in green) requires the action of the β-secretase, which render sAPPβ (soluble β-APP fragment) and βCTF (β-carboxi-terminal fragment) fragments; and γ-secretase, forming the sAPPγ (soluble γ-APP fragment) and γCTF (γ-carboxi-terminal fragment) fragments; while the action of α-secretase will avoid the neurotoxic pathway, producing the non-amyloidogenic fragments sAPPα (soluble α-APP fragment) and αCTF (α-carboxi-terminal fragment) (in blue).

The amyloid hypothesis, which is the generally accepted hypothesis for AD, suggests that the accumulation of β-amyloid peptide (Aβ) is the key mechanism causing the disease. The Aβ comprises 39–43 amino acids and is generated from the proteolysis of β-amyloid protein precursor (APP) [4]. This proteolysis is performed sequentially by the β-secretase and γ-secretase enzymes. Each secretase cleaves the protein at a different cleavage site producing different APP fragments including the soluble Aβ isoform which is the more neurotoxic form, whereas the insoluble form promotes the production of free radical and lipid peroxidation [5] (Figure 2). Therefore, β-secretase and γ-secretase mutations, which increase their enzymatic activity or mutations that raise APP expression, would be possible causes of the Aβ accumulation. These mutations are observed in the early-onset or familial AD (FAD), which is an autosomal-dominant genetic disease that appears in 30–50 year old patients [6]. FAD is caused by mutations in genes encoding APP or presenilins 1 (PS1) and 2, being the latter those that regulate secretases activity and, as result, APP proteolysis leading to Aβ formation. 

The amyloid cascade hypothesis proposes that Aβ excess, including the highly toxic Aβ oligomers, would lead to the formation of extracellular deposits, which are named amyloid or senile plaques. They would be preferably formed in the cerebral cortex and the hippocampus [6]. These plaques would provoke an inflammatory response through the activation of microglial cells and astrocytes, which would lead to the release of pro-oxidant and pro-inflammatory cytokines by these cells. The inflammation would produce neuronal damage and would alter neural homeostasis, which in turn would lead to hyperphosphorylation of the microtubule-associated protein Tau and its accumulation resulting in the appearance of intracellular neurofibrillary tangles. This would prevent Tau from its correct association with microtubules, modifying neuronal cytoskeleton and axonal transport, which might cause synapse dysfunction that is directly related to the cognitive deficit observed in AD patients [6].

### 2.2. Vascular Damage

The oxidative stress caused by aging and environmental or genetic factors, could also cause blood-brain barrier (BBB) dysfunction [7]. The BBB affectation would lead to the accumulation of neurotoxic serum protein in the brain that would produce inflammatory reactions and vascular damage and finally, it would result in the accumulation of Aβ and Tau proteins because of deficits in both their metabolism and clearance through neural vasculature. These pathological events would cause a cerebral amyloid angiopathy, which is associated with a higher risk of dementia because of the appearance of microaneurysms and small hemorrhages in the CNS [8].

### 2.3. Glial Cells Involvement in AD Pathophysiology

Inflammation, mediated by glial cells, plays an important role in AD pathophysiology [9] since AD patients show a chronic inflammatory reaction which may precede the neurodegeneration caused by Aβ [6]. In addition to producing an inflammatory response, glial cells are also responsible for the homeostasis of ions and neurotransmitters in the neuronal microenvironment. There are four groups of glial cells which could be involved in AD pathophysiology: microglial cells, oligodendrocytes, NG2 glial cells, and astrocytes [6]. The microglial cell activation found in proximity to senile plaques could be explained by the fact that the main functions of activated microglia are the phagocytosis of cell debris or foreign particles and cytokines released to fight CNS insults [10]. On the other hand, oligodendrocytes are responsible for providing the myelin sheaths of neuronal axons which allow the fast propagation of action potentials. In the vicinity of the senile plaques, myelin breakdown can be observed, and it is believed to be due to the iron released by oligodendrocytes which would promote Aβ oligomerization and deposition, potentiating Aβ toxicity [11]. The myelin breakdown and iron release is more common in late-myelinating regions and this fact may explain why certain brain areas are more affected by Aβ toxicity than others.

NG2 cells, also called oligodendrocyte progenitor cells (OPCs) because they can differentiate into oligodendrocytes, are the most recently discovered type of glial cells [12] being characterized by chondroitin sulphate proteoglycan-like immunoreactivity expression. NG2 cells are proposed to play a role in Aβ uptake and its degradation by the lysosomal pathway [13]. The number of NG2 cells has been found to be reduced in brains from AD patients [14] which might contribute to AD progression. Finally, astrocytes, the most abundant glial cells in the CNS, are responsible for several functions such as: elimination of neuronal debris, regulation of metabolism and energy supply in the brain, defense against oxidative stress through production of glutathione, prevention from neuronal excitotoxicity through glutamate levels regulation, and modulation of synaptic activity [15,16]. They contribute to preserving neuronal plasticity and remove neural waste proteins (such as Aβ and Tau) [15]. However, morphological alterations of astrocytes may be observed before senile plaques appear [17]. Moreover, Aβ accumulation would provoke reactive astrogliosis characterized by astrocyte proliferation and hypertrophy [8].

### 2.4. Oxidative Stress in AD

Oxidative stress is generated when the cellular antioxidant defense system is not able to cope with oxidant species. The reactive oxygen species (ROS) and reactive nitrogen species are the most common oxidant species and they can be physiologically formed in response to a pathogen attack or as products in metabolic reactions. Oxidant compounds, also named free radicals, own unpaired valence electrons which can react with many cell structures and molecules producing oxidation and deteriorating them.

Increased ROS production is usually caused by a mitochondrial dysfunction or chronic inflammatory responses, being both mechanisms involved in AD development [7]. Free radicals may be associated to early stages of AD and could be the trigger for Aβ accumulation, because oxidative stress increases β-secretase and γ-secretase activity, thus increasing Aβ formation. In addition, oxidative stress has been described to cause BBB impairment, which would also contribute to Aβ accumulation. Once senile plaques have been formed, they would provoke the activation of microglia cells and astrocytes, which ultimately would release pro-inflammatory and pro-oxidant cytokines and raise the amount of ROS, which would boost the disease progression [7]. 

### 2.5. Role of Actin Depolymerizing Factor (ADF)/Cofilin Rods in AD Pathogenesis

Apart from Tau-mediated cytoskeletal pathology, AD is associated to the presence of ADF and cofilin aggregates in the cerebral cortex and hippocampus. Cofilin and ADF are two proteins which play a key role in actin/cytoskeleton dynamics, but cofilin is notably more abundant than ADF that is why commonly the bibliography is focused on cofilin rather than on ADF. Cofilin can sever actin filaments but it also stabilizes short actin filaments by binding to F-actin and saturating it [18]. The active form of cofilin is required in dendritic plasticity because it is an actin binding protein that takes part in the synapses between dendrites and axons. However, when cofilin is activated it may form 1:1 cofilin-actin rods and disrupt cytoskeletal dynamics, impairing dendritic spine morphogenesis and synapse function [19]. Moreover, cofilin rods would obstruct neurites and would physically block axonal transport, avoiding the traffic of nutrients, proteins and organelles required for synaptic maintenance. All of this would contribute both to the neurite distal degeneration and the synapses damage observed in AD [20].

### 2.6. NMDAr Signaling and AD

NMDAr is an ionotropic receptor that requires the binding of glutamate, as agonist, jointly with glycine (or D-serine), as co-agonist, in order to be activated. Besides, its activation is voltage-dependent and cell depolarization is needed to release the Mg^+^ (magnesium) ion which is blocking the channel at resting membrane potential. These receptors are characterized by their diversity in molecular composition, differing the biophysical and pharmacological properties according to the subunits composition, as well as in its subcellular localization [21]. The pathological role of NMDAr in neurodegenerative diseases is mainly due to the excitotoxicity mediated by these receptors, caused by an excessive Ca^2+^ (calcium) influx in response to a chronic excess of glutamate levels by the diminished ability of astrocytes to uptake and metabolize glutamate, among other mechanisms leading to neuronal death [22]. It is suggested that the NR2B subunit-containing NMDAr has a crucial role in Aβ toxicity since the impairment of LTP (long-term potentiation) caused by Aβ oligomers exposure is reduced by the use of NR2B subunit antagonists (ifenprodil) [23].

## 3. The Delivery Challenge into the CNS

A common bottleneck for different compounds intended for therapeutic and/or diagnostic procedures for CNS diseases, including AD, is the limited access to the CNS caused by the presence of the BBB. Current drugs used in AD therapy (rivastigmine, galantamine, donepezil and memantine) are orally administered, which is more convenient for the patient but generates side effects as a result of its action on peripheral tissues due to the lack of selectivity for its therapeutic targets [24,25]. For instance, acetylcholinesterase inhibitors provoke nausea and vomiting which may lead to discontinuation of the treatment [26]. These obstacles might be solved by designing drug carriers capable of crossing the BBB and delivering the drug into the CNS at an effective dose and with minimum off-target effects. Nanoparticles (NPs) represent a very promising approach to facilitating BBB crossing and delivering diagnostic and/or therapeutic compounds into the brain.

### 3.1. BBB Crossing in AD

**Figure 3:**
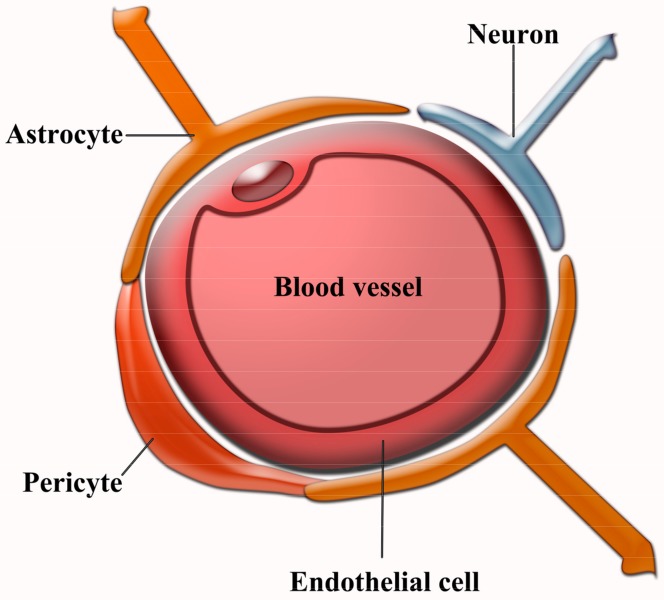
Cellular organization in the blood-brain barrier (BBB).

The BBB covers the CNS limiting the traffic of substances between the brain and the peripheral blood circulation. It maintains the required homeostasis of nutrients, glucose, and oxygen for neuronal survival and its proper functioning. The BBB is mainly composed by: endothelial cells, forming the cerebral microvascular endothelium; astrocyte endfeet, and pericytes, which work as nexus between endothelial cells and astrocytes (Figure 3). Endothelial cells are arranged forming tight junctions that prevent passive diffusion of both hydrophilic substances and those larger than 400 Da, which would need a specific carrier or receptor to cross the BBB by active transport. These cells also possess efflux transporters, such as P-glycoprotein (P-gp), ATP binding cassettes, and multidrug resistance-associated proteins, which reinforce the trafficking control of substances between the CNS and peripheral blood. The BBB is not only a physical barrier, but also a chemical one because of the different degrading and metabolizing enzymes that endothelial cells express, which, combined with the aforementioned, constitute a major obstacle for drug delivery into the brain [27]. A standard way of increasing BBB crossing by NPs is to use the mechanisms of transcytosis through the endothelial cells either through the adsorptive pathway used mainly by positively charged NPs or the, more specific, a receptor-mediated pathway that relies on the decoration of the NPs with ligands for receptors and transporters located on the luminal plasma membrane of the BBB’s endothelial cells. Several receptors and transporters have been used as targets for those ligands, such as the glucose transporter-1 (GLUT1) [28,29] or the transferrin receptor [30,31]. Another way to facilitate BBB crossing is to increase the NP circulation time since the longer the NP is in the bloodstream the more chances for the ligand coupled to the NP to interact with its target and to be internalized. The addition of polyethylene glycol (PEG; PEGylation) is the strategy most widely used to achieve this goal since it has been approved by the FDA and it is a safe mechanism to protect NPs from enzyme degradation, opsonisation, and phagocytosis by immune cells [30].

Another point that has to be taken into account is that in AD animal models some BBB dysfunction has been described. So, while under healthy conditions P-gp transporters contribute to Aβ peptide efflux from the brain, in AD the levels and activity of this transporter are decreased [32] which might contribute to Aβ peptide accumulation in the brain. In addition, more subtle BBB dysfunctions have been described in AD animal models such as decreased expression in the GLUT1 transporter, which might lead to a decrease in glucose uptake by the brain, that might limit some brain functions [33] or the known effect of Tau protein disrupting BBB integrity [34].

### 3.2. NPs for the Therapeutic and Diagnostic Compounds Delivery into the CNS in AD Animal Models

There are many types of NPs that have been devised to reach the CNS for different applications. Focusing on AD, NPs have been proposed both to treat and diagnose the disease. The therapeutic role may be achieved by the intrinsic properties of NPs and/or by their use as drug delivery systems designed to allow the access of certain drugs to their therapeutic targets inside the CNS by coating the NP surface with ligands which allow BBB crossing and CNS targeting as previously mentioned. The most relevant NPs related to AD are briefly described hereinafter.

#### 3.2.1. Lipid NPs (LNPs)

**Figure 4:**
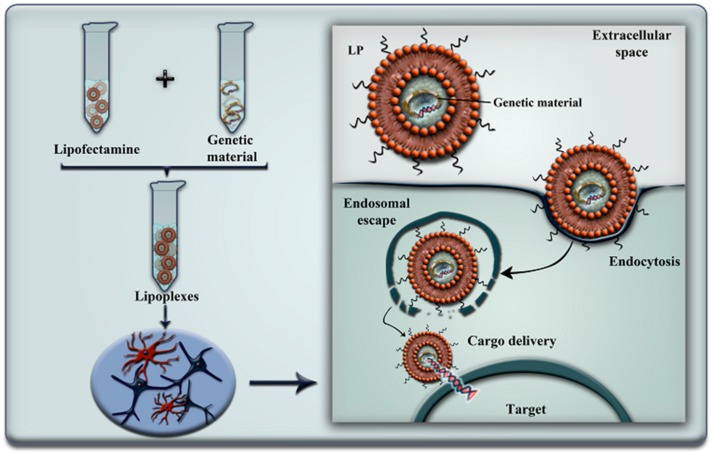
Scketch of Lipofectamin 2000 transfection. Lipofectamin 2000 is a commercial liposome (LP) which has been widely used as a genetic material carrier. Focusing on AD, Lipofectamin 2000 has been used as siRNA vehicle to reverse the neurotoxic effect of Aβ by disrupting the cofilin/RanBP9 pathway [37,38]. First, the lipoplex is formed by mixing Lipofectamine solution and the genetic material. Once the lipoplex has been coupled, it is administered to cell cultures and incubated as the manufacture’s protocol describes. Then, the lipoplex is internalized into the cell by endocytosis, thus it will perform endosomal escape before delivering its cargo into the cell.

Lipid-based NPs are widely used for delivering drugs and genetic material to different cell types due to their lipophilic nature. There are several types of lipid NPs including: lipid nanocapsules, liposomes (LP), solid lipid NPs (SLN), and stable nucleic acid lipid NPs. In this review, we will only refer to those lipid-based NPs that have been used for drug delivery in AD models. LPs are NPs composed of one or more lipid bilayers, which enclose an aqueous phase and thus isolate it from the external environment. This structure allows LPs to carry either lipophilic molecules into the lipid bilayers, or hydrophilic molecules into the aqueous core [35]. The delivery mechanisms performed by LPs are depicted in Figure 4. Moreover, cationic LPs would also be able to incorporate negatively charged molecules—such as genetic material—by electrostatic adsorption to the cationic lipids that form the bilayers. Apart from that, the LP surface could be modified by adding polymers, polysaccharides, or peptides in order to modulate certain characteristics of the NP. Thus, an LP could be decorated with an antibody providing targeting properties to the NP. Another widely used modification of LPs is PEGylation that would avoid the opsonization process, protecting the LP against the immune system and improving its pharmacokinetics and biodistribution [36].

Cationic LPs have been used to deliver drugs and genetic material more efficiently than neutral or anionic LPs. This is likely due to the fact that the positive charge of the NP would facilitate the interaction between the NP and the cell membrane and therefore would enhance the LP uptake. Nevertheless, reaching the CNS is more complex than that. Thus, a larger amount of cationic LPs would be needed to reach the CNS since the NP positive charge would interact with peripheral tissues and serum proteins, which would diminish the amount of LPs able to access the CNS [36]. LPs have been proposed as possible carriers for therapeutic compounds aimed to treat AD. Some of their applications are summarized in Table 1.

SLNs are generally composed of lipids, which remain in a solid state at room and body temperature. SLNs differ from LPs in their structure because SLNs lack the hydrophilic domain and therefore only lipophilic drugs can be transported by this type of NP. On the other hand, the lipophilic character of SLNs allows them to cross the BBB and remain in the CNS, being good candidates to efficiently deliver therapeutic and/or diagnostic compounds into the brain [39]. SLNs were modified including liquid lipids inside, giving way to the denominated nanostructured lipid carriers (NLCs), developed in order to improve their drug-loading ability and biocompatibility, being the higher loading capacity their main advantage over SLNs. However, NLCs are also composed of an aqueous phase which results in a lower stability because of the major susceptibility to bacteria growth [40].

#### 3.2.2. Dendrimers (DDs)

DDs are macromolecules formed by polymers organized in a geometrical shape. They are composed by three domains: the central core, the branches attached to the core as repetition units (their number would determine the DD generation) and the terminal functional groups which would interact with other molecules [39]. In addition to the basic structure, the DDs’ surface could be modified to provide them with additional properties. Thus, DDs’ decoration with transferrin would facilitate BBB crossing by the interaction with specific receptors located in the surface of BBB endothelial cells [41]. Moreover, PEGylation or the addition of carbohydrates would prevent toxic effects and provide a better stability in the biological milieu [42,43]. There are different DD types including, among others, poly-amidoamine (PAMAM), phosphodendrimers, poly-propylene imine, carbosilane, poly-l-lysine (PLL), and triazine. A comprehensive description can be found in the book by Tomalia et al. [44]. DDs have been used to transfect neurons in vitro [45] and it has been also described that cationic phosphorous DDs, at high concentrations, can disrupt Aβ and MAP-TAU (microtubule-associated protein-Tau) aggregation [46]. Moreover, a G0-PAMAM DD with tetra-maleimidopropionyl groups in its surface and decorated with helical β-peptide foldamers have shown protection against Aβ-induced long-term potentiation (LTP) impairment in mouse hippocampal slices, suggesting a protecting action against memory loss in AD [47]. DDs have been also used in animal models of AD for their intrinsic properties to prevent Aβ or α-synuclein fibrillation. So, G3/G4 polypropylenimine DDs with maltose groups in their surface were able to reduce the Aβ burden in APP/PS1 mice, a generally accepted model of AD (Table 1).

#### 3.2.3. Polymeric NPs (PNPs)

PNPs are NPs composed of polymers, such as poly-(butyl cyanoacrylate), poly-(lactic acid), poly-(lactic-*co*-glycolic acid) and also natural materials such as albumin and chitosan. The advantage of these compounds over the NP types described below is that they can be easier biodegraded and, therefore, they show minimal toxicity [48]. They can also form nanocapsules (spherical structures covered by a solid shell containing a liquid cavity) and nanospheres (spherical solid particles) [39] (Table 1). These NPs are also susceptible to surface modifications (such as PEGylation) which would improve both circulation lifetime and BBB penetration. PNPs can be loaded with either solid drugs or drug solutions remaining the loaded drug non-covalently absorbed or chemically bound to the NP surface. Despite of the good degradation rate of these NPs, the chronic character of neurodegenerative diseases and therefore the need of repetitive administrations of the therapeutic compound could lead to polymer accumulation that might cause undesirable toxicity [48].

#### 3.2.4. Magnetic NPs (MNPs)

MNPs are formed by a metal core which is usually composed by elements with unpaired electrons (Fe, Ni, Co, Cr or Gd) and is responsible for the magnetic properties. The most common cores used in nanomedicine are formed by iron oxides because it can be eliminated through the endogenous iron metabolic pathway and therefore causes lower toxicity. The iron oxide-state confers a better stability than the iron element. The core can be coated with different molecules, such as polysaccharides (dextran), polymers (PEG), phospholipids, peptides, etc. The coating is responsible for modulating its pharmacokinetic properties and toxicity, apart from protecting the magnetic core against the attacks of chemical species [49]. The MNP’s surface can be also decorated with fluorophores and/or radionuclides which would provide additional properties for image probes [50].

These type of NPs show theranostic properties, since they can be used for both diagnostic and therapeutic procedures, behaving as contrast agents for magnetic resonance imaging (MRI) and also as drug carriers. They are characterized by a good loading capacity, low toxicity, and magnetic properties which confer the ability to deliver the drug at specific sites in response to an applied magnetic field in addition to allow MRI. The magnetic NPs’ applications in AD treatment and diagnosis are summarized in Table 1.

#### 3.2.5. Gold NPs (AuNPs)

AuNPs owe their name to their gold core, which is usually covalently coated with an organic layer in order to provide better biophysical properties or targeting characteristics. Their optical and electric features allow them to adopt different structures such as nanospheres or nanorods [51]. They are also characterized by present a low toxicity, and be able to cross the BBB and reach CNS in a relatively easy way.

The use of AuNPs includes therapeutic aims such as drug carriers to deliver different cargos into the CNS, radiotherapy for tumors and photo-thermal therapy. They can also be used for imaging applications [52]. Some of the possible applications in AD animal models are reviewed in Table 1.

#### 3.2.6. Carbon Nanotubes (CNTs)

CNTs are formed by graphene sheets shaped as a cylinder with their ends opened. The cylinder can be made by a single graphene layer anchored by Van der Waals forces (single-walled), forming a flexible structure; or it can be formed by several grapheme layers (multi-walled), which will turn the structure into a less flexible one [39]. CNTs have been proposed for a wide spectrum of applications in the nanomedicine field. They may be functionalized with chemotherapy drugs or proteins bond to their surface. They could also be used for gene therapy serving as DNA carriers. On the other hand, their use as biosensors is noteworthy for diagnostic aims [53]. Their applications related to AD are described in Table 1.

## 4. Conclusions

AD is a complex disease that lacks a curative treatment. Actual therapeutic options only delay disease progression. For this reason, effective therapies capable of reversing the cognitive damage that has been already caused are needed. There is still a long way to go to define the causal nexus between the observed physiopathological changes in the brain and the appearance of the dementia syndrome. Nevertheless, the knowledge collected about AD pathophysiology over many years, gives way to promising lines of research despite the ambiguity that surrounds the original cause of the disease. Meanwhile, one of the problems that the therapies of CNS diseases face is BBB crossing which severely limits the access of many drugs into the brain. NPs are promising tools to overcome the limitations that BBB generates for many therapeutic and diagnostic compounds to access the CNS. Summing up, although promising results of the NPs’ use in both cellular and animal models of AD have been reported, a clear pathway leading to a cure for this disease remains elusive. This is mainly due to the lack of identification of a single cause for AD which translates into different hypothesis trying to explain the origin of this disease. The cause of the origin of AD has been identified only in the familial forms of the disease where specific mutations lead to an accumulation of Aβ peptide. However, familial forms of AD represent a small percentage of all AD patients.

## Figures and Tables

**Table 1:** The use of nanoparticles (NPs) applied to AD treatment and/or diagnosis.

NP	Core/Type	Surface Ligands	Cargo	Applications in Ad	Ref.
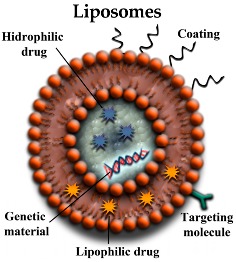	LP (PC + CH)	PEG	Galantamine	Allowed intranasal administration of galantamine, which improved its pharmacodynamic and pharmacokinetic properties.	[54]
	Lipofectamine 2000^®^	Unknown	Cofilin siRNA	Reversed mitochondrial superoxide production and Ca^2+^ deregulation mediated by cofilin in response to Aβ stimulation.	[37,38]
	LP (PC + CH)	CPP + PEG	Rivastigmine	Improved rivastigmine distribution in hippocampus and cortex by intranasal administration compared to free drug and intravenous administration. Also diminished adverse effects.	[55]
	Nano-LP (DSPC + CH)	TrF-mAb + PEG	Curcumin	Retardation of Aβ aggregation. Could be used for Aβ plaques labelling due to the affinity between curcumin and Aβ peptide.	[56]
	LP (SPG + CH)	Phosphatidic acid/Cardiolipin	None	Reduced Aβ peptide amount in the plasma in a rodent model which may modify Aβ levels in the brain.	[57]
	LP (SPG + CH)	Phosphatidic acid + ApoE	None	Increased Aβ clearance from the brain.	[58]
	Nano-LP (SPG + CH)	RI-OR2-TAT + Maleimide-PEG	None	Inhibited the formation of Aβ oligomers and fibrils in vitro, reduced activated microglial cells, and increased the number of neurons.	[59]
	LP (DSPC + CH)	PEGOX26 mAb, 19B8MAb	None	LPs coupled with OX26 mAb, through the streptavidin-biotin complex, were able to reach the rat brain after tail vein injection.	[60]
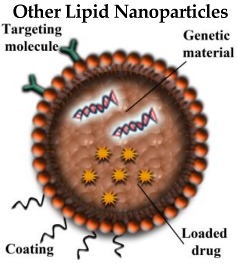	Piperine SLN	Polysorbate 80	Donepezil	Improved cognitive function and diminished Aβ plaques and tangles.	[61]
	NLC (pεC, CTG)	Polysorbate 80	Indomethacin	The encapsulation of indomethacin allowed a higher drug concentration in brain, which results in improved behavior in rats after Aβ injection. This seems to be due to a reduction of microglial activation.	[62]
	NLC(LDL-mimic)	PEG + Lactoferrin	Curcumin	Targeted brain tissue and reduced malondialdehyde levels (indicator of oxidative stress) compared to curcumin solution.	[63]
	SLN	Pluronic	Galantamine	Improved memory process compared to free drug.	[64]
	SLN	Not specified	Chrysin	Restores lipid peroxidation and acetylcholine esterase activity that were increased after Aβ administration.	[65]
	SLN	OX26 mAb	Resveratrol	Targeted the BBB and prevented Aβ peptide fibrillation.	[66]
	SLN	CPP (RVG-9R) + Chitosan	BACE1 siRNA	Diminished Aβ peptide burden by silencing of β-secretase protein.	[67]
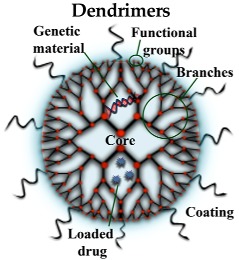	G3/4-CPD	Not specified	None	Disrupted Aβ and MAP-TAU aggregation at high concentrations and accelerated fibrils formation at low concentrations.	[46]
	G3-GATG	Morpholine groups	None	Accelerated Aβ aggregation preventing the toxic effects of immature amyloid fibrils, which are more harmful than mature fibrils.	[68]
	G0-PAMAM	Tetra-maleimidopropionyl + Helical β-peptide foldamers	None	Protective effect against Aβ-induced LTP impairment.	[47]
	G3/4-PPI	Maltose/maltotriose	None	Maltose DDs reduced Aβ burden in APP/PS1 mice, while cationic maltose DDs provoked memory loss in wild-type mice.	[42]
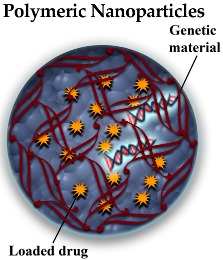	Chitosan	Polysorbate 80	Tacrine	Provided a diffusion-controlled release of the drug.	[69]
	Chitosan	Not specified	Rivastigmine	Improved rivastigmine bioavailability and uptake in brain through intranasal administration.	[70]
	PLGA	Not specified	Curcumin	Reduced learning and memory impairments Aβ-induced through activation of Wnt/β-catenin pathway, which increases neurogenesis.	[71]
	PLA	PEG + TGN + OSH	None	NP was capable of target Aβ peptide and had low toxicity which suggested this NP as a possible vehicle to be used in AD treatment.	[72]
	Chitosan	Polysorbate 80	Galantamine	Allowed intranasal administration of galantamine improving its brain uptake.	[73]
	P(HDCA-*co*-RCA-*co*-MePEGCA)/P(MePEGCA-*co*-Bio-PEGCA-*co*-HDCA)	PEGRhodamine/BiotinAβmAb	None	Tg2576 mice were intravenously injected with the NPs, resulting in improved results in the Novel Object Recognition test, which were similar to wild type mice. Although a low diffusion into the brain was found.	[74]
	PLGA	Not specified	Tarenflurbil	Improved pharmacokinetics and oral bioavailability compared to free tarenflurbil and could allow intranasal administration.	[75]
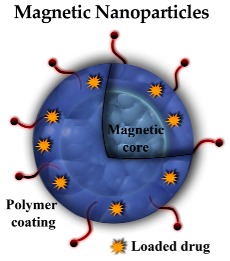	NIF-maghemite (Fe_2_O_3_)	AβmAb clone BAM10	None	Detection (MRI and FI ex vivo) and disruption of Aβ fibrillation.	[76]
	Magnevist^®^ (Gd-DTPA)	IgG-antiamyloid antibody + Chitosan + 125I	CTX	Contrast imaging of cerebrovascular amyloid (MRI, SPECT). Diminished pro-inflammatory cytokine compared with free cyclophosphamide.	[77]
	Magnetite (Fe_3_O_4_)	PEG/PVP + Curcumin	None	Detection of amyloid plaques by MRI.	[78]
	Magnetite (Fe_3_O_4_)	AβOmAb + Nitro-DOPA + PEG	None	Detection of Aβ oligomers as an early AD biomarker (MRI).	[79]
	AGuIX^®^ (Gd^3+^)	KLVFF/LPFFD + PEG + Cyanine 5.5	None	Selectively target Aβ1-42 fibrils and detects senile plaques (MRI).	[80]
	Magnetite (Fe_3_O_4_)	AβpAb/APPpAb	None	Imaging of Aβ plaques (MRI).	[81]
	Iron oxide (not specified)	DSPE-PEG-NHS + Congo Red	Rutin	Congo Red: detected senile plaques by specifically bind to Aβ; Rutin: Interfered with Aβ aggregation and neurotoxicity, is anti-inflammatory and antioxidant.	[82]
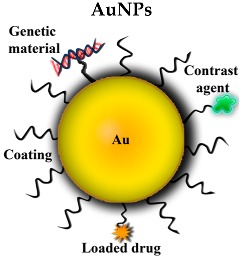	Au	Carboxyl-conjugated AuNPs (negative charged)	None	Disrupted Aβ fibrillation and fragmented the fibrils already formed.	[83]
	Au	Cu^2+^:PEI/Hemin:PEI	None	Colorimetric detection of monomeric Aβ peptide (dual recognition: AuNP:PEI:Cu^2+^-Aβ-Hemin:PEI:AuNP).	[84]
	Au	Nanorods associated to CLPFFD or CTAB	None	Aβ detection and reduction of amyloidogenic process by NIR irradiation.	[85]
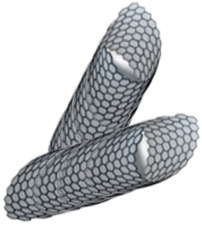	SWCNT	Not specified	Ach	Allows Ach delivery in the brain tissue.	[86]
	SWCNT	Not specified	None	Prevents β-sheet formation by destabilization of prefibrillar β-sheet (shown by computational study).	[87]
	SWCNT	Cr/Au + linker + Aβ antibody	None	Construct of CNT-MESFET devices for Aβ peptide detection.	[88]
	SWCNT	Not specified	None	Restores normal autophagy by depressing mTOR activity and reversing lysosomal proteolytic dysfunction.	[89]
	MWCNT	Secondary mAbTAU	None	Use as mass enhancers in a classic sandwich assay for TAU immuno-detection.	[90]

**Abbreviations:** AβmAb = Aβ monoclonal antibody; AβOmAb = Aβ oligomers monoclonal antibody; AβpAb = Aβ polyclonal antibody; Ach = acetylcholine; ApoE = apolipoprotein E-derived peptide; APPpAb = APP polyclonal antibody; BACE1 = β-site amyloid protein precursor cleavage enzyme 1; CH = Cholesterol; CLPFFD = Aβ-binding peptide; CPD = cationic phosphorous dendrimers; CPP = cell penetration peptides; CTG = capric/caprylic triglycerides; CTAB = cetyltrimethylammonium bromide; CTX = Cyclophosphamide; DSPC = 1,2-distearoyl-sn-glycero-3-phosphocholine; DSPE-PEG-NHS = 1,2-dioleoyl-sn-glycero-3-phosphoethanolamine-n -[poly(ethylene glycol)]-hydroxy succinamide; FI = fluorescence imaging; G0/1/2/3/4 = generation 0/1/2/3/4; GATG = gallic acid-triethylene glycol) dendrimers; KLVFF, LPFFD = small peptides derived from the sequence of Aβ1-42; mAbTAU = TAU 12 clone; MRI = magnetic resonance imaging; mTOR = protein kinase mammalian Target of Rapamycin; MWCNT = multi-walled carbon nanotube; NIF = near-infrared fluorescent; NLC = nanostructured lipid carrier; NP = nanoparticle; OSH = Aβ-binding peptide (QSHYRHISPAQV); OX26 mAb = OX26 monoclonal antibody (bind to BBB cells that express transferrin receptors); PAMAM = poly-amidoamine; PEG = polyethylene glycol; PEI = polyethyleneimine; pεC = poly(ε-caprolactone); P(HDCA-*co*-RCA-*co*-MePEGCA) = Poly[(hexadecyl cyanoacrylate-*co*-rhodamine B cyanoacrylate-*co*-methoxypoly(ethylene glycol cyanoacrylate)]; PLA = poly(lactic acid); PLGA = poly(lactic-*co*-glycolic acid); P(MePEGCA-*co*-Bio-PEGCA-*co*-HDCA) = poly[methoxypoly(ethyleneglycol) cyanoacrylate-*co*-Biotin-poly(ethylene glycol) cyanoacrylate-*co*-hexadecyl cyanoacrylate]; PPI = poly-propylen-imine; PVP = polyvinylpyrrolidone; RI-OR2-TAT = Retro-inverso peptide; RVG-9R = rabies virus glycoprotein of 9 arginine residues; SLN = solid lipid nanoparticle; PC = Phosphatidyl-choline; SPECT = single photon emission computed tomography; SWCNT = single-walled carbon nanotube; TGN = targeting peptide to overcome BBB (TGNYKALHPHNG); TrF-mAb = Anti-transferrin monoclonal antibody.
